# Childhood Obstructive Sleep Apnea Associates with Neuropsychological Deficits and Neuronal Brain Injury

**DOI:** 10.1371/journal.pmed.0030301

**Published:** 2006-08-22

**Authors:** Ann C Halbower, Mahaveer Degaonkar, Peter B Barker, Christopher J Earley, Carole L Marcus, Philip L Smith, M. Cristine Prahme, E. Mark Mahone

**Affiliations:** 1 Department of Pediatrics, Johns Hopkins University School of Medicine, Baltimore, Maryland, United States of America; 2 Department of Radiology, Johns Hopkins University School of Medicine, and FM Kirby Center for Functional Brain Imaging, Kennedy Krieger Institute, Baltimore, Maryland, United States of America; 3 Department of Neurology, Johns Hopkins University School of Medicine, Baltimore, Maryland, United States of America; 4 Department of Pediatrics, University of Pennsylvania, Philadelphia, Pennsylvania, United States of America; 5 Department of Medicine, Johns Hopkins University School of Medicine, Baltimore, Maryland, United States of America; 6 Department of Neuropsychology, Kennedy Krieger Institute, Baltimore, Maryland, United States of America; 7 Department of Psychiatry, Johns Hopkins University School of Medicine, Baltimore, Maryland, United States of America; Sleep Medicine Research Center, United States of America

## Abstract

**Background:**

Childhood obstructive sleep apnea (OSA) is associated with neuropsychological deficits of memory, learning, and executive function. There is no evidence of neuronal brain injury in children with OSA. We hypothesized that childhood OSA is associated with neuropsychological performance dysfunction, and with neuronal metabolite alterations in the brain, indicative of neuronal injury in areas corresponding to neuropsychological function.

**Methods and Findings:**

We conducted a cross-sectional study of 31 children (19 with OSA and 12 healthy controls, aged 6–16 y) group-matched by age, ethnicity, gender, and socioeconomic status. Participants underwent polysomnography and neuropsychological assessments. Proton magnetic resonance spectroscopic imaging was performed on a subset of children with OSA and on matched controls. Neuropsychological test scores and mean neuronal metabolite ratios of target brain areas were compared.

Relative to controls, children with severe OSA had significant deficits in IQ and executive functions (verbal working memory and verbal fluency). Children with OSA demonstrated decreases of the mean neuronal metabolite ratio N-acetyl aspartate/choline in the left hippocampus (controls: 1.29, standard deviation [SD] 0.21; OSA: 0.91, SD 0.05; *p* = 0.001) and right frontal cortex (controls: 2.2, SD 0.4; OSA: 1.6, SD 0.4; *p* = 0.03).

**Conclusions:**

Childhood OSA is associated with deficits of IQ and executive function and also with possible neuronal injury in the hippocampus and frontal cortex. We speculate that untreated childhood OSA could permanently alter a developing child's cognitive potential.

## Abstract 2

Childhood obstructive sleep apnea is associated with deficits of IQ and executive function and also with possible neuronal injury in the hippocampus and frontal cortex.

## Editors' Summary

**Background.**

Sleep is essential for health, and in children it is crucial to normal development. Symptomatic childhood sleep-disordered breathing (SDB) is the name for a range of conditions in which children have difficulties with breathing when they are asleep. The conditions range from simple snoring to the most severe condition, known as obstructive sleep apnea (OSA). Apnea means a temporary absence of breathing, and in OSA this is caused by a temporary but repeated blockage of the flow of air to the lungs. In children, OSA occurs for a number of reasons including enlarged tonsils, long-term allergy, and obesity. About two in every hundred children have OSA. The symptoms of OSA are loud snoring at night, disrupted, restless sleep, undue tiredness, and difficulties in concentration. The main test for it is a sleep study (polysomnography). If untreated, researchers believe that it may lead to a number of long-term problems with health and learning; children with disorders of sleep have been shown to have memory problems, lower general intelligence, and worse executive function (the ability to adapt to new situations), and may have behavioral problems similar to those of attention deficit hyperactivity disorder (ADHD).

**Why Was This Study Done?**

Adults with sleep apnea have been shown to have abnormalities of parts of their brain, specifically the frontal cortex, cerebellum, and hippocampus, but so far there are no data on whether there are similar changes in children. Children with sleep apnea may have cognitive deficits, but the research on this topic is limited.

**What Did the Researchers Do and Find?**

The researchers wanted to investigate the brains of children with OSA to see if there was any evidence of changes in the brain and if these changes were associated with any learning problems. They studied 31 children (19 with OSA and 12 healthy controls, aged 6–16 y). Participants underwent polysomnography and neuropsychological assessments, such as IQ tests and tests of their ability to perform tasks involving decision making. Some of the children also had specialized scans of their brains (known as proton magnetic resonance spectroscopic imaging) that can measure the levels of certain metabolites—substances that are produced as a result of brain activity. The researchers then compared the neuropsychological test scores with the levels of the metabolites. They found that relative to controls, children with severe OSA had lower IQ and ability to perform tasks involving decision making. Children with OSA also had changes in metabolites in the brain similar to those seen in diseases in which there is damage to brain cells.

**What Do These Findings Mean?**

It seems clear that OSA in children is associated with learning problems, and that these learning problems may in turn be associated with changes in brain metabolites. The changes in metabolites are not necessarily permanent—in other diseases where changes have been found they can be reversed with treatment. If these results are confirmed in other children with OSA, it will highlight the importance of treating children for OSA as soon as possible. In addition, the measurement of metabolites may be a way of measuring how well children are responding to treatment.

**Additional Information.**

Please access these Web sites via the online version of this summary at http://dx.doi.org/10.1371/journal.pmed.0030301.

- MedlinePlus's encyclopedia has an entry on sleep apnea
- The American Sleep Apnea Association has information about having a child investigated for sleep apnea
- The National Sleep Foundation also provides information about sleep disorders

## Introduction

Individuals require a wide range of cognitive skills in order to function in society, so if the acquisition of these skills is perturbed during development, there may be a long-term effect on cognitive and psychological function. Obstructive sleep apnea (OSA)—defined as obstructed breathing efforts during sleep [1] with resultant gas exchange abnormalities and sleep fragmentation [2]—has been linked to increased cardiovascular mortality, increased automobile accidents, and cognitive function impairments in adults. Untreated childhood OSA enormously increases health resource utilization [3], and has been associated with growth problems, cardiovascular consequences, and neuropsychological dysfunctions such as learning and memory problems [4,5], decreased attention, and poor school performance [6]. From a cognitive standpoint, adult OSA has been linked to deficits of executive function (flexible adaptation to novel situations with an organized, goal-directed approach) [7]. In children, gains of executive function skills occur during developmental periods corresponding to the neuronal myelination and maturation of the prefrontal cortex [8,9]. Executive function is considered critical for school-age children to develop complex problem solving [10] and perform other volitional tasks in response to new situations with demands on working memory [11]. If untreated OSA causes neuropsychological or executive dysfunction in developing children, and if these skills are permanently impaired before maturation of the prefrontal cortex, it could severely alter a child's cognitive potential, ultimately impacting both the child's health and his or her functioning level in society.

Symptomatic childhood sleep-disordered breathing (SDB), which includes simple snoring and labored breathing, and partial obstructions that might not meet the adult criteria for OSA [12,13], has long been associated with behavioral dysfunctions including aggression, impulsivity, hyperactivity, and decreased attention based on subjective data provided by parents or teachers [14–21]. These behaviors mimic those associated with attention deficit hyperactivity disorder (ADHD), a disorder that presents with alterations in executive function [22]. Objective measurements of specific neuropsychological performance deficits related to sleep problems in children have been limited; however, recent studies have begun to identify significant differences in cognitive function between children with SDB and healthy controls. For example, Gottlieb and colleagues found significantly lower performance on measures of memory, executive function, and general intelligence in 5-y-old children with symptoms of SDB than in asymptomatic children [23]. O'Brien et al. demonstrated decreased general intelligence, language, and visual–spatial skills in 87 habitually snoring children [24]. O'Brien's group demonstrated even more significant neuropsychological deficits in children with more severe apnea [25].

The mechanisms causing these neuropsychological deficits have not been fully delineated. While sleepiness and sleep fragmentation might be readily reversible with treatment, neuronal injury resulting from long-term oxygen saturation abnormalities might represent a more pervasive health risk. There is evidence of altered brain function associated with blood gas abnormalities. Patients with sleep apnea have reduced cerebral blood flow and altered cerebrovascular responses to hypercapnia [26,27]. Hypoxia causes neuronal injury in vulnerable parts of the brain, especially the cerebellum and hippocampus, where the formation of lactate and free radicals is thought to lead to cellular injury [28,29]. The hippocampus is critically involved in learning and memory. Sleep deprivation in rats alters the synaptic plasticity of the hippocampus [30], and impairs hippocampus-mediated contextual learning [31] and spatial learning [32]. Intermittent hypoxia also causes spatial learning deficits and increased motor activity in juvenile rats [33]. The mechanism proposed for the spatial learning deficits observed in rats involved apoptosis of subpopulations of hippocampal neurons [34]. The authors suggested that the behaviors demonstrated by rats exposed to intermittent hypoxia were similar to those demonstrated by children with sleep apnea.

Imaging studies of adults with sleep apnea have identified abnormal morphology of the frontal cortex, cerebellum, and hippocampus [35,36]. Functioning in a complex integrated network, these brain areas are important for executive function, motor regulation of breathing [35,37], and memory function, respectively. Altered central nervous system metabolites of neuronal white and gray matter in OSA patients have been demonstrated using proton magnetic resonance spectroscopy imaging (MRSI). The authors suggested that hypoxemia resulting from sleep apnea might have caused cerebral neuronal injury. There is recent evidence that suggests a link between hippocampal metabolite alterations and deficits of cognitive function in adults with OSA [38].

These studies provide evidence that SDB is associated with observable reductions in cognitive function in both adults and children. In adults with sleep apnea, there are measurable morphological abnormalities in the brain, but adults with OSA often suffer from comorbid health problems such as diabetes [39], hypertension, and cardiovascular disease [40], which could confound the association. There have been no studies demonstrating such neuronal injury in children with sleep apnea. These studies are crucial, since childhood OSA impacts a rapidly developing brain, and thus the long-term consequences of neuronal injury may be far greater than those seen in adults.

The purpose of this study was to examine the neuropsychological deficits associated with moderate to severe childhood sleep apnea, and to determine whether these deficits are associated with neuronal changes in vulnerable target areas of the brain. We hypothesized that SDB would be associated with neuropsychological dysfunction in the areas of executive function, learning, and memory; and that central nervous system metabolite alterations would be observed in brain regions associated with these functions, i.e., the hippocampus and frontal cortex.

## Methods

### Design

This study was approved by the Western Institutional Review Board in collaboration with the Johns Hopkins University Internal Review Board. All researchers adhered to the Johns Hopkins University policies on patient privacy and research ethics in compliance with the Declaration of Helsinki. All parents or guardians signed informed consent forms for their children to participate in the research project, and children over the age of 8 y signed informed assent forms.

This was a cross-sectional study of participants aged 6–16 y with moderate to severe OSA compared to non-snoring healthy children group-matched by age, ethnicity, gender, and socioeconomic status (SES). OSA patients were identified by polysomnographic sleep studies performed at the Johns Hopkins sleep lab. All tests were performed while the OSA patients were awaiting surgical management. OSA participants met enrollment criteria if they had moderate to severe OSA by our definition, i.e., an apnea hypopnea index (AHI) ≥ 8 (see “Polysomnography” for definition of apnea). All participants underwent polysomnography and a battery of neuropsychological tests. Proton MRSI of the brain was performed on a subset of OSA participants and control children (those who met inclusion criteria and could tolerate imaging time without sedation).

#### Recruitment.

Normal healthy non-snoring participants had no known medical problems, and were recruited from inner-city Baltimore by advertisements placed in the Johns Hopkins Hospital (downtown campus), in public sites in the inner city, and in a local African-American newspaper (since >70% of our clinic patients are African-American). For matching of SES, children were recruited from Baltimore City, with confirmation of zip code, maternal education level, and Hollingshead index. Hollingshead index is an index of SES based on parent education and type of job. Guardians of normal children were questioned by phone to determine eligibility for inclusion, with extensive questions about past medical history, medication use, and demographic information. At the time of the neuropsychological testing, questionnaires were used to verify history, medications, and demographic information.

The age range for this study was selected based on several criteria. The selected neuropsychological tests have been standardized and validated in this age range. Furthermore, there is a track record of success of brain imaging in children of this age range at our center, without the need for behavior training or sedation. Additionally, normal neuronal metabolite concentrations in the brain have been established for children in this age range [41–43]. Imaging studies in childhood OSA have not been performed previously, therefore we chose children with severe sleep apnea to more likely identify possible neuronal injury associated with OSA.

#### Exclusionary criteria.

Exclusionary criteria included a full scale IQ score ≤ 75 on initial testing, neurological abnormalities revealed by history or radiological or electroencephalogram (EEG) studies, use of psychotropic or sedative medications for the last 2 wk before study, or non-English speakers because of the difficulty of performing neuropsychological tests through an interpreter. Children were also excluded if there was a history of significant medical, psychiatric, vision, or hearing impairment, or mental retardation. Children with life-threatening sleep apnea requiring emergency management were also excluded. Children with other sleep problems such as insomnia, parasomnias, bedtime behavior difficulties, restless legs syndrome, or abnormal movement during sleep were excluded. Children with sleep apnea who had a previous diagnosis or symptoms of ADHD or a history of chronic hyperactivity (*n* = 5) were not excluded because of the common overlap of these disorders with OSA; however, data analyses were performed both with and without these children for comparison. Furthermore, the children with OSA and ADHD were assessed only while off psychotropic medication for more than 2 wk. All participants were screened to ensure that they had no contraindications for MRI, namely claustrophobia, cardiac pacemaker, orthodontics (braces), or other non–magnetic-resonance-compatible surgical/ferromagnetic implant. If contraindications were noted, children were allowed to proceed with neuropsychological testing but MRI was not performed. Normal control children were not excluded for ADHD if they otherwise met criteria (healthy, non-snoring, no other exclusion criteria or psychotropic medications). Control children with subsequent findings of AHI >1, or prolonged hypercapnia (>50 mm Hg for >20% of total sleep time) measured by polysomnogrpahy were excluded, and there was no crossover to the OSA group.

### Polysomnography

Polysomnography was performed on all participants. During the sleep study, surface electrodes and monitoring devices measured signals from central EEG, right and left electro-oculogram, surface EMG, ECG, chest and abdominal wall motion, and end-tidal PCO_2_ (Novametrix, Wallingford, Connecticut, United States). Pulse oximetry with an 8-s averaging time was used to record the time in minutes of any oxygen saturation less than 95% to detect brief oxygen saturation changes (Masimo, Irvine, California, United States). Airflow was measured by oro-nasal thermistor in all children. Additionally, nasal pressure was monitored in order to obtain a more quantitative flow signal (Protech, Mukilteo, Washington, United States). Polysomnogram data were displayed digitally (Alice 4, Atlanta, Georgia, United States). All studies were monitored with real-time video for motion analysis and snoring recording.

Respiratory parameters of interest included arousals, apneas, and hypopneas, low oxygen saturation time (SaO_2_T), hypercapnia time, and oxygen saturation nadir (SaO_2_N). The arousal index (AI) was the number of arousals and awakenings measured by a shift of EEG signal to the alpha or beta range for greater than 3 s (as previously defined by the American Sleep Disorders Association [44]) divided by the total sleep time in hours. An apnea was defined as an absence of airflow for two or more breath cycles. Hypopnea was a visible decrease in airflow by nasal pressure signal (or by thermistor when pressure signal was unavailable) and either an EEG arousal or a drop in oxygen saturation of 3% or greater. A mixed apnea was an obstructive apnea in combination with a central (absent effort) apnea. The AHI comprised the obstructive, mixed, and hypopnea events divided by total sleep time in hours; central apneas were not included in the AHI. SaO_2_T was the time (in minutes) with oxygen saturations less than 95% (in order to detect mild intermittent desaturations), and SaO_2_N was the severity of oxygen desaturation. Hypercapnia time was the time in minutes that the end-tidal CO_2_ monitor detected a CO_2_ level greater than 50 mm Hg. There is no established definition of mild, moderate, or severe sleep apnea in children. OSA in adults is defined by the AHI: the number of apnea or hypopnea (partial obstruction) events per hour. However, continuous partial obstruction is underestimated by these criteria, and is considered significant by the Johns Hopkins Sleep Program. The AHI in normal non-snoring children has been determined to be less than 1.0 with little hypercapnia (time with CO_2_ > 50 mm Hg) [45–47]. For this protocol, mild OSA was defined as AHI 1–5, moderate OSA as AHI 5–10, and severe OSA as AHI greater than 10.

### Neuropsychological Evaluation

The neuropsychological assessment was administered by a psychometrician (M. C. P.) supervised by a licensed psychologist (E. M. M.), both of whom were masked to the group assignment of the participants at the time of testing. The protocol was selected to include areas considered to be vulnerable in children with OSA—executive function and memory—as abnormalities of these brain systems may negatively impact psychosocial function and have been shown to be affected in adults with sleep apnea [48]. In addition, domains hypothesized to be less affected in children with OSA (visual–spatial perception and motor speed) were also included for comparison. Global intelligence was measured using the full scale IQ scores from *WISC-III* [49] (*n =* 15) or *WISC-IV* [50] (*n =* 16). In addition to IQ, the assessment protocol included measures of executive function (i.e., response preparation, inhibition, and working memory), attention, verbal and visual memory, neuromotor function, cerebellar function (i.e., perceptual and motor timing), and visual–spatial perception.

### Magnetic Resonance Spectroscopy

Magnetic resonance spectroscopy detects steady state levels of native metabolites present in the brain. Among the brain compounds easily measured with magnetic resonance spectroscopy are N-acetyl aspartate (NAA), choline (Cho), and creatine (Cr, often used as a background comparison).

Children were introduced to the MRI machine at the FM Kirby Center for Functional Brain Imaging by use of a full-size mock scanner where the protocol and noise could be demonstrated ahead of time. The children were asked to lie still and they were supported with foam pillows to reduce movement. They were able to listen to music or watch movies with specially designed headphones and mirrors inside the actual scanner. No sedation was used in any study.

**Figure 1:**
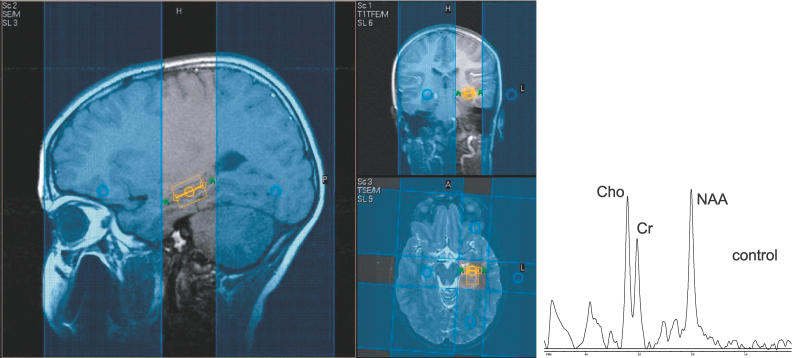
Single Voxel Image from the Left Hippocampus in an 11-y-Old Normal Male Child Blue areas are outer volume suppression bands that improve the accuracy of the hippocampal signal by suppressing surrounding lipid signal. On the right is a spectroscopy signal from a 11 year old male child, the spectrum demonstrating peaks from NAA, Cho, and Cr.

Two different approaches were used for proton MRSI of the brain: multi-slice MRSI for coverage of supratentorial brain regions, and single voxel methods for the hippocampus and cerebellum. All magnetic resonance examinations were performed on a 1.5 Tesla clinical system (Philips Intera NT, Phillips Medical Systems, Best, Netherlands). Routine T1 and T2 weighted MRI sequences were used to screen and exclude for any structural abnormalities. Three oblique axial slices (thickness, 15 mm; intersectional gap, 2.5 mm) parallel to the anterior commissure–posterior commissure line were selected for the proton MRSI. Outer volume saturation pulses were used for suppression of lipid and water signals originating from the skull and scalp, and a chemical-shift-selective saturation pulse was used for water suppression. The three interleaved slices were recorded with a repetition time (*T*_R_) of 1,700 ms and an echo time (*T*_E_) of 280 ms (field of view, 24 × 18 cm; matrix size, 32 × 24; signal average, 1), giving a total of 15 min for data acquisition with circular *k*-space sampling. The nominal voxel size was 0.8 cm^3^. Spectra were evaluated from the following brain regions: putamen, thalamus (predominantly pulvinar region), dorsal parietal cortex, parietal white matter, frontal white matter, medial premotor cortex, and frontal cortex. In order to minimize partial volume effects, only voxels that appeared completely encompassed by the boundaries of the anatomic regions of interest were included. Single voxel methods utilized a PRESS sequence with a short echo time (*T*_R_/*T*_E_ 1,500/35 ms) placed in the body of the left hippocampus and the mesial left cerebellum. Voxel size was 2 × 1.5 × 1.5 cm. For both spectroscopic methods, metabolites were expressed as ratios in comparison to levels of other metabolites, specifically, the NAA/Cr, Cho/Cr, and NAA/Cho ratios. This measurement method effectively detects metabolite changes that move in opposite directions such as the decreased NAA to increased Cho seen in ischemic brain injury [51] and in the injury reported in adult OSA [52]. Figure 1 shows an example of single voxel imaging of the hippocampus in a normal male child, with blue outer volume suppression bands used to improve the accuracy of the hippocampal signal by suppressing surrounding lipid signal.

### Statistical Analysis

#### Neuropsychological testing.

For neuropsychological testing, the primary predictor variable was group status (control versus OSA measured by the AHI). Secondary predictor variables were other polysomnographic parameters (AI, SaO_2_T, SaO_2_N, and CO_2_ > 50) and body mass index (BMI). Primary outcomes were mean standard neuropsychological test scores. Levene's test was used to ensure homogeneity of variance between groups. The Mann–Whitney *U* statistic for nonparametric analysis was used for all variables in which the Levene's test was significant. Analysis of variance was used to determine differences in the outcomes assessed as a function of group status. *T-*tests were performed assuming equal variance, and the two-tailed significance is reported. Group differences were also compared by effect size, measured by eta squared (η^2^), a measure of contrast between groups independent of sample size [53]. According to Cohen [54], values of 0.01, 0.06, and 0.14 are used to indicate small, medium, and large associations between variables, respectively.

#### Brain Magnetic Resonance Spectroscopy.

For brain imaging studies, sleep apnea patients were compared to group matched normal children where the outcome variable was mean cerebral metabolite ratios determined by measurement of mean cerebral metabolite ratios of target areas of the brain on MRSI. *T-*tests were performed assuming equal variance, and the two-tailed significance is reported.

#### Sample size and statistical power.

Previous reports of cognitive dysfunction in severe childhood OSA demonstrated significant effects when comparing healthy controls to as few as five OSA patients and nine snoring patients without OSA [55]. Power analysis was conducted to determine the appropriate sample size needed in each group in order to detect group differences between the sleep apnea patients and normal participants on mean neuropsychological test scores. Using the criteria of a power of 0.80, a type I error rate of 0.05, a mean test–retest reliability of selected instruments of 0.80, and estimates of moderate to large effect size (i.e., η^2^ > 0.07), based on our preliminary data, it was determined that a sample size of 13 participants in each group would be adequate to detect group differences in neuropsychological test score results between sleep apnea patients and normal participants. Considering the multiple comparisons, only neuropsychological test results with a significance level *p* < 0.05 and η^2^ > 0.1 were considered significant and interpreted further in our analyses to control for both type I and type II error. MRSI has not been previously performed in children with OSA; however, adults with OSA demonstrated differences in hippocampal metabolites with eight patients versus five controls [38]. Since this study represents the first MRSI in children with OSA, we performed brain imaging on all eligible children who could tolerate the procedure. Correction for multiple comparisons was not made, in order to gather data for future study.

## Results

### Demographics

Thirty-four children were initially enrolled after screening for exclusionary criteria, but two did not return for testing, and one control was excluded after a finding of snoring and prolonged end-tidal CO_2_ levels greater than 50 mm Hg (358 min) despite a normal AHI and oxygen saturation. Demographic information for the entire eligible sample (*n =* 31) is listed in Table 1. There were no significant group differences in SES, measured by median household income, or maternal education between groups. Additonally, the Hollingshead index, an index of SES based on parental education and job type, was not different between the groups. There were no significant differences between the two groups in age, gender ratio, handedness, or racial distribution (see Table 1). Of the 19 OSA patients, five had a history of hyperactivity or diagnosis of ADHD, and analyses are presented separately for OSA and OSA excluding ADHD (“OSA/not ADHD”) groups. No normal children with ADHD answered the recruitment advertisements, therefore the control group does not include any individuals with ADHD. After excluding the children with ADHD (*n =* 5) from the OSA group, there were still no differences between groups in age, SES, gender ratio, handedness, or racial distribution. The individuals in the OSA groups had significantly higher BMIs than did controls.

**Table 1:**
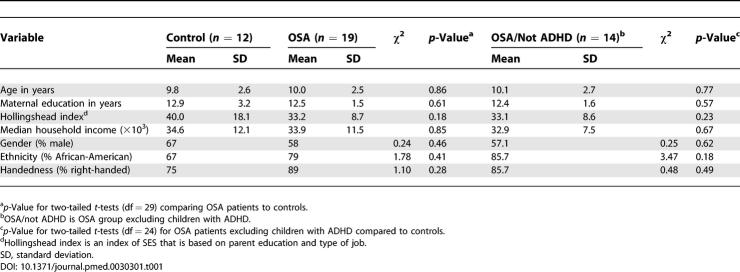
Demographic Parameters for Entire Sample (*n =* 31)

### Polysomnography Results

Respiratory parameters for OSA and control groups are listed in Table 2. The mean and median AHI in the OSA group fell into the severe range by our criteria, although there was a large standard deviation in time with oxygen saturation less than 95% (SaO_2_T; 0–271 min), SaO_2_N (48%–96%), and time with CO_2_ levels greater than 50 mm Hg (0–371 min).

**Table 2:**
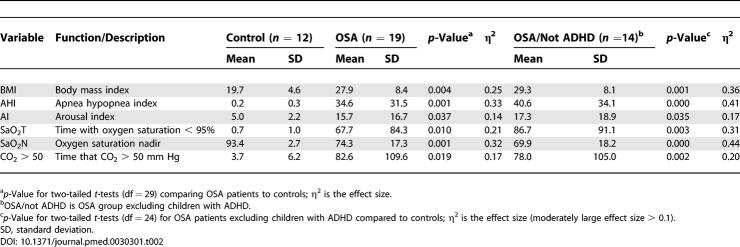
Respiratory Parameters for Entire Sample (*n =* 31)

### Neuropsychological Function

Results of neuropsychological test performance for all groups are listed in Table 3. Children with OSA had significantly lower scores than matched controls on full scale IQ. The OSA group also had significantly lower performance on measures of executive function, including verbal working memory (sentence span) and word fluency (measured by the Delis-Kaplan Executive Function System (D-KEFS) category fluency test). In contrast, the executive functions reported to be impaired in adult sleep apnea [5,56,57] (i.e., problem solving and planning, inhibitory control, sustained attention, and vigilance) were not affected in children with severe sleep apnea. There were no significant differences between groups on any of the other neuropsychological variables; however, both visual–spatial memory (Children's Memory Scale Dot Locations, η^2^ = 0.10) and verbal memory (California Verbal Learning Test for Children, η^2^ = 0.09) demonstrated large effect sizes, suggesting that, with a larger sample, statistically significant differences between groups on these measures might be detected. Motor speed, perceptual timing, and motor timing (dependent on cerebellar functioning) were also not affected in our full sample of sleep apnea patients; however, when the children with ADHD were excluded from the OSA group, there was a significant deficit noted on the perceptual timing task (η^2^ = 0.29, *p* < 0.05). The group differences in full scale IQ, verbal working memory, and word fluency remained significant after the children with ADHD were excluded. BMI was noted to correlate with decreased IQ (*r* = −0.45, *p* = 0.019); however, when BMI was controlled for the AHI (after confirming BMI overlap between the groups), this effect was no longer significant (*r* = −0.32, *p* = 0.106).

**Table 3:**
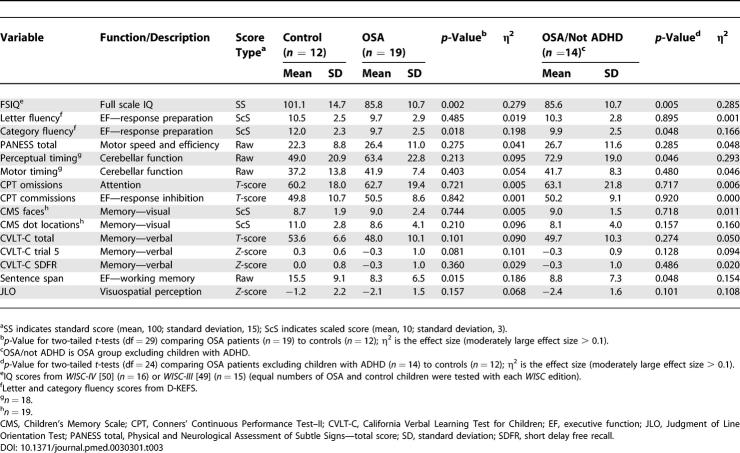
Neuropsychological Assessment Data for Entire Sample

### Proton MRSI

Proton MRSI studies of the brain were performed in a subset of children with OSA and controls. Children with ADHD were analyzed and reported separately (see Tables 4 and 5; the OSA group excluding ADHD are labeled as “OSA/not ADHD” in the tables). Of the 31 eligible children successfully completing a polysomnogram, 26 underwent some or all of the brain imaging studies. Dropouts or exclusions from brain imaging included the following: one with metal dental work and four who could not tolerate the MRI (three obese children complained of claustrophobia). Regions of interest in the brain scan corresponded to areas hypothesized to be linked to neuropsychological deficits, including white and gray matter of the frontal and parietal lobes, assessed by MRSI, and the middle left hippocampus and cerebellum, assessed by the single voxel method. The total scan time was 1 h. The children were not sedated; therefore, some regions of interest were unable to be analyzed because of motion artifact (*n* varies depending on the image reported).

**Table 4:**
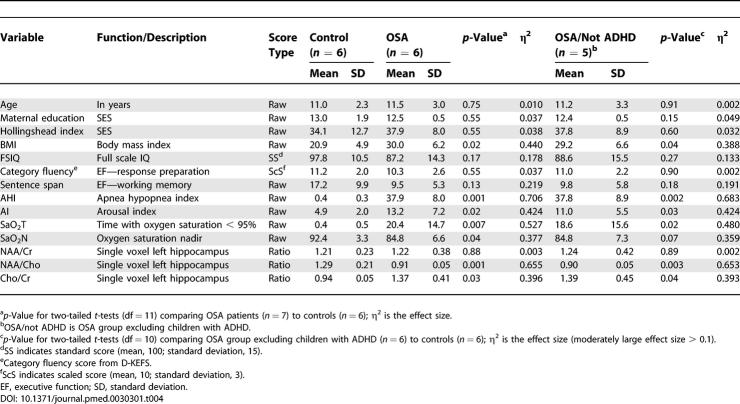
Left Hippocampal Neuronal Metabolites in OSA Compared to Control Children

**Table 5:**
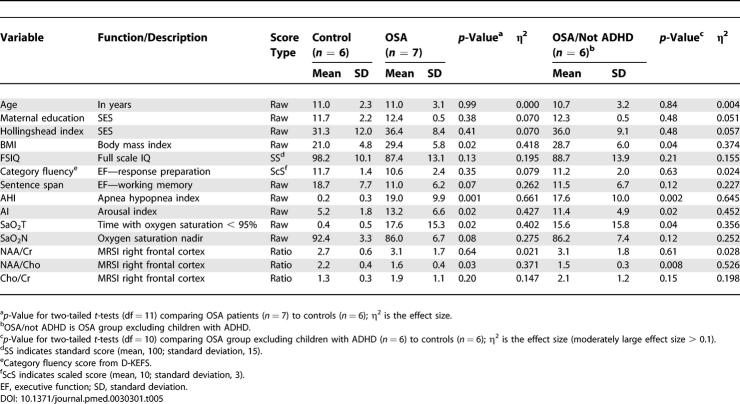
MRSI of Right Frontal Cortex: OSA Patients Compared to Controls

Single voxel spectroscopy measurements of the body of the left hippocampus were analyzed in 12 children (six patients with OSA and six controls; see Figure 1), and results are reported in Table 4. Within this subsample, there were no significant differences between groups in age, maternal education, gender distribution (χ^2^ = 4.4, *p* = 0.08), handedness (χ^2^ = 0.1, *p* = 0.65), or race (χ^2^ = 0.02, *p* = 0.73). The group differences in neuropsychological test scores in this subsample continued to show a large effect size. Compared to controls, the OSA group had a significant decrease in hippocampal NAA/Cho (*p* = 0.001) and a significant increase of Cho/Cr (*p* = 0.03), indicating abnormal neuronal metabolism in the static state.

**Figure 2:**
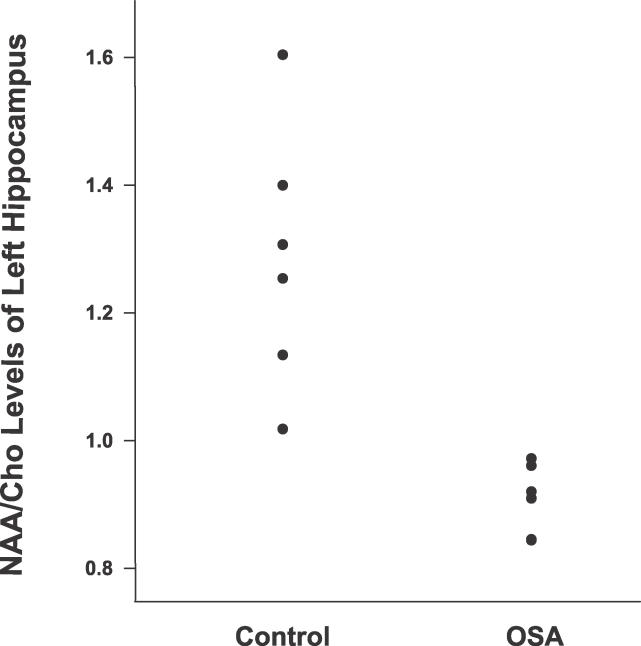
Hippocampal Neuronal NAA/Cho Metabolites Are Decreased in Children with OSA aged 9–16 y The ratio of NAA to Cho is decreased in the hippocampus of OSA patients compared to normal children.

These differences remained when the subsample was analyzed excluding the child with ADHD from the OSA group. There was little overlap of results between the two groups in this subsample aged 9–16 y (see Figure 2).

Only two children under age 9 underwent single voxel MRSI of the hippocampus (one 7-y-old in each group; the control was male and the OSA patient was female). The OSA patient had an AHI of 33.8, but her NAA/Cr ratio demonstrated an unusual value (2.16), more than twice that of all the other OSA patients, and significantly higher than that of all the normal children (range of NAA/Cr levels for all OSA and control participants: 0.89–1.98). When results were compared including these younger children, the increased Cho/Cr in the OSA group remained significant (*p* = 0.05), while NAA/Cho was no longer significantly decreased because of the unusual value of NAA/Cr in the OSA patient driven by the increased NAA spectral peak (see Discussion).

MRSI of cortical structures was performed in 13 children (seven OSA patients and six controls), and results are presented in Table 5. Within this subsample, there were no significant differences between groups in age, maternal education, gender distribution (χ^2^ = 1.6, *p* = 0.23), handedness (χ^2^ = 0.1, *p* = 0.57), or race (χ^2^ = 1.4, *p* = 0.51). There continued to be a large effect size difference in neuropsychological test scores between groups. Metabolite ratios were compared between groups in frontal and parietal white and gray matter, as well as thalamus, putamen, and premotor cortex. There was a significant difference between groups in the NAA/Cho ratio in right frontal cortex (*p* = 0.03). This difference was even more significant when we excluded children with ADHD from the OSA sample (*p* = 0.008). There were no significant group differences (regardless of ADHD status) in the other brain areas measured using MRSI.

Single voxel MRSI studies of the cerebellum in 12 children (five controls and seven OSA patients) showed no differences between groups on cerebellar metabolite ratios: Cho/Cr (*p* = 0.53, η^2^ = 0.04), NAA/Cr (*p* = 0.61, η^2^ = 0.03), or NAA/Cho (*p* = 0. 24, η^2^ = 0.14), although given the large effect size for NAA/Cho, the difference might be significant with a larger sample.

## Discussion

To our knowledge, this is the first study utilizing MRSI to measure central nervous system metabolites in children with OSA, and to identify central nervous system changes associated with neuropsychological dysfunction in childhood OSA. This study demonstrated two primary findings: (A) neuronal metabolites in pediatric OSA patients were altered in the hippocampus and the right frontal cortex, indicating possible neuronal injury linked to severe childhood OSA, and (B) children with severe OSA had significantly lower IQ and executive control functions compared to normal children matched for age, gender, ethnicity, and SES. These cognitive functions depend on neuronal networks corresponding to the structures where we identified metabolite alterations.

In the current study, children with sleep apnea and neuropsychological dysfunction demonstrated decreased levels of NAA/Cho in both the hippocampus and the right frontal cortex. The brain areas affected are important in IQ and executive function and corroborate the findings from morphological studies of adult OSA showing decreased gray matter in the same regions [35,36]. While these findings do not necessarily indicate a causal relationship between OSA and neuronal abnormality (i.e., it is not known whether sleep apnea causes brain damage or pre-existing brain damage causes sleep apnea), it will be important to determine to what extent these findings reverse with treatment. The NAA signal localizes predominantly to neurons, axons, and dendrites within the central nervous system. Decreased NAA signal is notably seen in diseases with neuro-axonal loss or dysfunction [58,59], while increases in Cho occur in pathological conditions during active demyelination, as well as with changes of membrane metabolism or glial cell reaction [60,61]. Normal changes in Cho are also seen during myelin maturation in early development [41], but tend to be stable after age 2 y [43]. Previous animal studies of irreversible cerebral ischemia demonstrated decreased NAA signals by proton MRSI within minutes of injury, and these metabolite signals were detected prior to changes noted by typical magnetic resonance imaging [51]. NAA/Cho alterations are not necessarily permanent, as noted in studies of childhood encephalopathy or acute multiple sclerosis where reduced NAA/Cho improved after resolution of illness [62,63]. Therefore, dysfunction or acute injury to the axon can lead to decreased NAA prior to axonal loss. It is not clear, however, whether long-standing metabolite alterations are reversible, or whether there is a vulnerable age of neuronal plasticity that leads to permanent brain injury with neuronal metabolite alterations [64,65].

The laterality of our findings to the right frontal cortex is interesting, particularly given the neuropsychological findings involving verbal working memory and fluency, thought to lateralize to the left frontal cortex. Executive functions are supported by a distributed neural network with cortical and subcortical components including the frontal cortex and its striatal-thalamic-cerebellar connections [7,11,57,66]. Volumetric studies of adults with OSA demonstrate diffuse gray matter loss bilaterally in the frontal lobes, but those participants may have had long-standing OSA [35]; therefore, our finding in children may reflect changes earlier in the course of disease.

The neuronal metabolite levels of NAA/Cho in the hippocampus were significantly reduced in children with OSA aged 9–16 y compared to controls, as demonstrated in Figure 2, where there is virtually no overlap between the two groups. We identified a younger child (a 7-y-old female with OSA) with unusually high NAA/Cr and NAA/Cho levels driven by a high NAA spectral peak. There are regional variations of NAA and Cho concentrations in the brain, and NAA/Cho levels are known to increase with age, especially between birth and age 2 y, but increases are subtle after age 2, up to a peak at age 10 y [43]; therefore, the elevated NAA/Cho in the 7-y-old OSA patient would not be an expected age-related change [41,43,67,68]. An increase in NAA is associated with increasing axon density, increased dendrites, and increased synaptic connections [41]. In rats exposed to intermittent hypoxia, apoptosis of hippocampal neurons correlating with spatial memory dysfunction was later reversed by neurogenesis, which is a unique capability of the hippocampus [33]. Additionally, gender-specific protection from brain injury in females [69] has been seen in experiments using intermittent hypoxia, which caused decreased branching dendrites in male but not female rats during development. The increased NAA spectral peak noted in the 7-y-old female with OSA might be an outlier, or might represent neuronal recovery and neurogenesis in the damaged hippocampal neurons as seen in the animal studies. This finding requires validation with young participants in order to determine whether certain age groups or genders are more susceptible to neuronal injury or recovery.

The findings of decreased IQ and altered executive function demonstrated by our study suggest that OSA can impact a child's ability to learn and adapt to new challenges, or to perform in school. The participants in our study had severe sleep apnea. Although the prevalence of severe childhood sleep apnea is not known, 17% of otherwise healthy children aged 6–16 y referred to our clinic from the local primary care providers for symptoms of SDB fall into this category. The neuropsychological deficits in this study of children with severe sleep apnea were more profound than those found in previous studies of mild OSA. However, these results corroborate the findings of several pediatric studies of cognition in childhood SDB, where deficits in general intelligence [23,25,55,70–73] and some measures of executive function [23,25,74] were noted. Compared to previous findings in adults with OSA, some executive functions (i.e., problem solving, planning, inhibitory control, and sustained attention) were not affected in children with severe sleep apnea [5,75]. Motor speed was also not affected in our sleep apnea patients.

Unlike most other pediatric studies of neuropsychological deficits linked to mild or moderate SDB, we enrolled children with severe sleep apnea from a lower socioeconomic area with lower maternal education. The IQ levels were more profoundly impacted in our children with OSA compared to previous pediatric studies, with the exception of the Montgomery-Downs study of at-risk children [70], the O'Brien study of significant sleep apnea in children with a minority population of 60% (mean AHI 9.8) [25], and the Rhodes study of teenagers where the participants were morbidly obese [55]. These studies demonstrated that multiple risk factors may be additive in terms of neuropsychological consequences. In contrast to our study, the neuropsychological differences between apnea and control populations in the vast majority of pediatric studies may be underestimated because of enrollment of affluent populations with either high maternal education or high average IQ levels (conferring a possible protective effect on measurable neuropsychological deficits) [15,21,23,71,74,76], or because of design limitations such as the inclusion of children with snoring or mild OSA (AHI 1–5) in the control group [23,55,76] or the lack of confirmation of normal breathing in control groups defined with a polysomnogram [15,21,73,74,77].

The OSA participants in our study were significantly more overweight or obese than the control participants. This study limitation reflects the reality of our referral population, in which more than 40% of our general pediatric clinic patients are reported to be at risk for obesity. We were unable to recruit enough non-snoring obese controls to match for obesity, and snoring controls were excluded because of previous studies demonstrating that snoring is not normal [24]. Most studies focusing on the neuropsychological impacts of childhood sleep apnea do not include information on the BMI of the children enrolled. Obesity is a risk factor for SDB and is a common finding in children referred to sleep centers [78]. Both SDB and obesity can affect quality of life [79,80]. If obesity and SDB interact, these combined problems as well as lifestyle factors linked with obesity (such as television time) may play an important role in exacerbating neuropsychological impairments associated with sleep apnea.

We admitted into our study five children with OSA who had symptoms or a diagnosis of ADHD given the common overlap of sleep disturbances and daytime symptoms in children with ADHD and with OSA [81]. It is difficult to distinguish ADHD as a premorbid versus comorbid feature associated with sleep apnea. Likewise, many children seen in the clinic have symptoms of hyperactivity or ADHD but have not undergone formal diagnostic testing, therefore underestimating the prevalence of a comorbid disorder. It has been previously shown that executive functions are impaired in children with ADHD [82], especially notably in children with average or less than average IQ levels [83]. When children with ADHD were excluded from the OSA population, the significance of our findings did not change, indicating that they were not influencing our results.

In clinical studies of OSA, it is difficult to differentiate the effects of sleep fragmentation from hypoxemia on neuropsychological function, since by definition the AHI encompasses both sleep fragmentation and oxygen saturation abnormalities. This study was not designed to separate these factors. Although the magnitude of the association of respiratory parameters to cognitive deficits was stronger than that of the AI, the AI might play a significant role in cognitive deficits with a larger sample. Several pediatric studies of snoring children without gas exchange abnormalities (often termed “primary snoring”) demonstrate effects on attention [24,71,72,74], IQ [23,24,71,72], or memory [23,72], indicating a possible role for sleep fragmentation or sleep disruption in neuropsychological dysfunction. The participants in our study were older, with more severe apnea, than the participants in studies of primary snoring, suggesting that age or severity of OSA may contribute to the relative neuropsychological impacts of sleep fragmentation versus oxygen deprivation injury. There may be a spectrum of SDB, from sleep fragmentation, to mild disease, to more severe disease. However, chronic sleep disruption during active brain development could potentially lead to permanent decreased cognitive potential, thus causing as serious a situation as oxygen deprivation to the brain.

We were limited to the number of participants available in this study because of the expense of brain imaging. We acknowledge that some comparisons may have shown statistically significant group differences with larger samples, and effect sizes are reported to assist in the interpretation of effects for future research. For example, cerebellar neuronal abnormalities associated with childhood OSA and related neuropsychological dysfunction (e.g., perceptual timing) may indeed be observed in larger samples.

Childhood OSA has a prevalence of about 2% in the general population [84,85], and severe sleep apnea cases represent 17% of the otherwise normal school-aged children referred to our sleep center. OSA in adults has been linked to increased cardiovascular morbidity and mortality, increased automobile accidents, and cognitive function impairments. We found that childhood OSA is associated with deficits of IQ and executive function and also with abnormal neuronal metabolites in the hippocampus and frontal cortex, indicating possible neuronal injury. We speculate that untreated childhood OSA could permanently alter the trajectory of a developing child's ultimate cognitive potential, resulting in a lifetime of health and economic impacts. It remains to be determined if early identification and treatment can reverse the neuronal and performance deficits identified in this study of childhood OSA. Future studies will need to address the effect of treatment, and explore gender- and age-related differences in vulnerability to help target early diagnosis and treatment most effectively.

## Supporting Information

Alternative Language Abstract S1 Translation of Abstract into Portuguese by Author (25 KB DOC)

Alternative Language Abstract S2 Translation of Abstract into French by Author (22 KB DOC)

Alternative Language Abstract S3 Translation of Abstract into Spanish by Author (22 KB DOC)

Alternative Language Abstract S4 Translation of Abstract into Tagalog by Author (22 KB DOC)

## Abbreviations

ADHD: attention deficit hyperactivity disorder
AHI: apnea hypopnea index
AI: arousal index
BMI: body mass index
Cho: choline
Cr: creatine
D-KEFS: Delis-Kaplan Executive Function System
EEG: electroencephalogram
MRSI: magnetic resonance spectroscopy imaging
NAA: N-acetyl aspartate
OSA: obstructive sleep apnea
SaO_2_N: oxygen saturation nadir
SaO_2_T: oxygen saturation time
SD: standard deviation
SDB: sleep-disordered breathing
SES: socioeconomic status
η^2^: eta squared
