# Supplementary material for: Childhood Obstructive Sleep Apnea Associates with Neuropsychological Deficits and Neuronal Brain Injury
Source: PLoS Med. 2006 Aug 22;3(8):e301. doi: 10.1371/journal.pmed.0030301 (PMC1551912; doi:10.1371/journal.pmed.0030301)
Supplement: Supplementary file 1 [file pmed.0030301.sd001.doc]

Abstract:

Childhood obstructive sleep apnea (OSA) is associated with neuropsychological deficits of memory, learning, and executive function. There is no evidence of neuronal brain injury in children with OSA. We hypothesized that childhood OSA is associated with neuropsychological performance dysfunction, and with neuronal metabolite alterations in the brain, indicative of neuronal injury in areas corresponding to neuropsychological function. Methods: Cross-sectional study of 31 children (19 with OSA and 12 healthy controls, aged 6-16 years) group-matched by age, ethnicity, gender, and socioeconomic status. Participants underwent polysomnography and neuropsychological assessments. Proton magnetic resonance spectroscopic imaging was performed on a subset of children with OSA and matched controls. Neuropsychological test scores and mean neuronal metabolite ratios of target brain areas were compared. Findings: Relative to controls, children with severe OSA had significant deficits in IQ and executive functions (verbal working memory and verbal fluency). Children with OSA demonstrated decreases of the neuronal metabolite ratio N-acetyl aspartate/Choline in the left hippocampus (p= 0.001) and right frontal cortex (p = 0.03). Conclusion: Childhood OSA is associated with deficits of IQ and executive function and also with possible neuronal injury in the hippocampus and frontal cortex. We speculate that untreated childhood OSA could permanently alter a developing child’s cognitive potential.

Resumo:

Apnéia obstrutiva do sono na infância é associada com defeitos neupsicologicos relacionados à memória, aprendizado e funções executivas. No entanto, no presente momento, não há evidência de lesão cerebral em pacientes com apnéia obstrutiva do sono. Nós formulamos a hipótese de que a apnéia obstrutiva do sono na infância é associada com disfunção neuropsicológica e alterações metabólicas neuronais no cérebro, indicativas de lesões neuronais em áreas com funções de controle neuropsicologico. Metodos: o estudo foi do tipo “cross sectional” e envolveu 31 crianças (19 com apnéia obstrutiva do sono e 12 controles normais, variando de 6 a 16 anos de idade) que foram pareadas por idade, etnicidade, sexo e nível socioeconômico. Os participantes tiveram avaliação polissonografica e neuropsicológica. Um subgrupo de crianças com apnéia obstrutiva do sono e controles normais foram avaliados por espectroscopia de ressonância magnética por próton. Testes neuropsicológicos e níveis de metabólitos cerebrais foram comparados nos 2 grupos. Resultados: Quando comparados aos controles, o grupo com forma severa da apnéia obstrutiva do sono apresentou déficits significativos de inteligência cerebral e funções cerebrais superiores (memória verbal e fluência verbal). Crianças com apnéia obstrutiva do sono mostraram reduções da relação dos metabólitos neuronais N-acetil aspartato/Colina no hipocampo esquerdo (p=0.01) e no córtex frontal direito (p=0.03). Conclusão: Apnéia obstrutiva do sono na infância é associada com possível lesão neuronal do hipocampo e córtex frontal. Nós especulamos que se não tratada, a apnéia obstrutiva do sono na infância pode alterar permanentemente o potencial de aprendizado da criança afetada.
