# Supplementary material for: Childhood Obstructive Sleep Apnea Associates with Neuropsychological Deficits and Neuronal Brain Injury
Source: PLoS Med. 2006 Aug 22;3(8):e301. doi: 10.1371/journal.pmed.0030301 (PMC1551912; doi:10.1371/journal.pmed.0030301)
Supplement: Supplementary file 2 [file pmed.0030301.sd002.doc]

**Propos :** L’apnée obstructive du sommeil (AOS) est associée chez l’enfant ā des troubles neuropsychologiques tels des problèmes de mémoire, d’apprentissage et de fonctions exécutives. Il n’y a cependant pas de données démontrant la présence de lésion cérébrale nerveuse chez les enfants atteints d’AOS. Nous avons émis l’hypothèse que l’AOS chez l’enfant serait associée à une dysfonction de la performance neuropsychologique avec altération des métabolites neuronaux cerebraux indicatifs de lesions neuronales dans des zones spécifiques de la fonction neuropsychologique. **Méthodes :** Etude de 31 enfants (19 souffrant d’AOS et 12 contrôles sains, agés de 6-16 ans) dont les groupes sont appariés en fonction de l’âge, l’ethnicité, le sexe, et le statut socio-économique. Les participants furent soumis à un polysomnogramme et à des tests neuropsychologiques. Des images spectrométriques du proton par résonnance magnétique furent obtenues chez un sous-groupe d’enfants atteints d’AOS et contrôles appariés. Nous avons aussi comparé les scores des tests neuropsychologiques et les valeurs moyennes des rapports metaboliques neuronaux de regions cérébrales spécifiques. **Résultats :** En comparaison au groupe de contrôle, les enfants souffrant d’AOS sevère ont montré des déficits significatifs du quotient intellectuel (QI) et des fonctions de l’exécution (mémoire de

travail verbale et fluidité verbale). Les enfants atteints d’AOS ont egalement demontré des réductions du rapport des métabolites neuronaux acide N-acetyl aspartique/Choline au niveau de l’hippocampe gauche (P = 0.001) et du cortex frontal droit (P = 0.03). Conclusion : L’AOS chez l’enfant est associée à des deficits du QI et des fonctions exécutives avec des lésions nerveuses possibles de l’hippocampe et du cortex frontal. Nous spéculons que l’OAS non traitée de l’enfant pourrait altérer de façon permanente le potentiel cognitif en développement de l’enfant.
