# Supplementary material for: Childhood Obstructive Sleep Apnea Associates with Neuropsychological Deficits and Neuronal Brain Injury
Source: PLoS Med. 2006 Aug 22;3(8):e301. doi: 10.1371/journal.pmed.0030301 (PMC1551912; doi:10.1371/journal.pmed.0030301)
Supplement: Supplementary file 3 [file pmed.0030301.sd003.doc]

**Antecedentes:** El síndrome de apnea obstructiva del sueño (SAOS) en niños se asocia con déficits neuropsicológicos en las areas de memoria, aprendizaje y funciones ejeciutivas. Sin embargo, no hay evidencia de lesion neuronal en el cerebro de los niños con SAOS. Nuestra hipótesis de trabajo es que el SAOS en niños se asocai con disfunción neuropsicológica debida a alteraciones en el metabolismo neuronal de las áreas cerebrales correspondientes. **Método.** Estudio transversal de 31 niños (19 con SAOS y 12 controles sanos, edad 6-16 años) de edad, etnia, sexo y estatus socio-económico similar. Entodos ellos se realizó un estudio polisomonográfico y valoración neuropsicológica. Se obtuvieron imágenes de espectroscopia por resonancia magnética de protones en un subgrupo de niños con SAOS y sus correspondientes controles sanos. Se compararon los resultados de los pruebes neuropsicológicos y los valores medios de los metabolitos neuronales en las areas cerebrales diana. **Resultados:** Comparados con los controles sanos, los niños con SAOS grave presentaban déficits significativos en el IQ y en diversas funciones ejeciutivas (memoria de trabajo verbal y fluidez de lenguaje). Los niños con SAOS presentaban disminución de cociente metabolico neuronal N-acetil aspartato/Cholina en el hipocampo izquierdo (*p*= 0.001) y en el cortex frontal derecho (*p* = 0.03). **Conclusión:** El SAOS en niños se asocia a déficits de IQ y función ejecutiva y también a posibles lesiones neuronales en el hipocampo y cortex frontal. Sugerimos que el SAOS no tratado en niños puede alterar de forma permanente el desarrollo de su potencial cognitivo.
