# Supplementary material for: Childhood Obstructive Sleep Apnea Associates with Neuropsychological Deficits and Neuronal Brain Injury
Source: PLoS Med. 2006 Aug 22;3(8):e301. doi: 10.1371/journal.pmed.0030301 (PMC1551912; doi:10.1371/journal.pmed.0030301)
Supplement: Supplementary file 4 [file pmed.0030301.sd004.doc]

**Kaalaman:**

Ang pambatang Obstructive Sleep Apnea (OSA) ay iniuugnay sa kakulangang *neuropsychological* ng alaala, pag-aaral, at mga gawain sa pagganap (executive function).  Walang katibayan ng *neuronal* na pinsala sa utak sa mga batang may OSA.  Teorya namin na ang pambatang OSA ay kaugnay ng hindi maayos na gawaing *neuropsychological*, kasama ng pagbabago ng *neuronal metabolite* sa utak. Ito ay nangangahulugan ng pinsalang *neuronal* sa mga bahaging nakatalaga para sa mga gawaing *neuropsychological*.

**Pamamaraan:**

Isang pagsusuri ang ginawa sa 31 bata, edad 6- 16. 19 sa kanila ang may OSA at ang natitirang 12 bata ay walang sakit. Ang mga bata ay magkatugma ayon sa edad, lahi, kasarian at katayuan sa buhay. Ang mga kasali sa pag-aaral ay sumailalim sa pagsusuring *polysomnography* at *neuropsychological*.

Isang grupo ng mga batang may OSA at katulad na batang walang sakit ay ginamitan ng *Proton Magnetic Resonance Spectroscopic Imaging*. Ikinumpara ang resulta ng pagsusulit *neuropsychological* at ang katampatan (mean) ng *neuronal metabolite ratio* sa mga piling bahagi ng utak.

M**ga natuklasan:**

Kung ikukumpara sa mga batang walang OSA, ang mga batang malala na ang OSA ay may makahulugang pagkukulang sa IQ at mga gawaing tagapagpaganap (executive function) kasama na ang paglalarawan sa alala (verbal working memory) at pagiging matatas sa pagsasalita (verbal fluency).  Ang mga batang may OSA ay nagpakita ng mababang *neuronal metabolite ratio N-acetyl aspartate/Choline* sa kaliwang *hippocampus* (p = 0.001) at kanang *frontal cortex* (p = 0.03).

Konklu**syon:**

Ang pambatang OSA ay iniuugnay sa pagkukulang sa IQ at mga gawaing tagapagpaganap (executive function). Maaari rin itong mangahulugan ng posibleng pinsala sa *hippocampus* at *frontal cortex*.  Sa palagay namin, kung hindi malulunasan ang pambatang OSA, maaaring tuluyang maiba ang *cognitive potential* ng isang lumalaking bata.
